# A Case of Systemic AL Amyloidosis Diagnosed by Screening Colonoscopy

**DOI:** 10.1155/2022/5562281

**Published:** 2022-04-20

**Authors:** Lynna Alnimer, Ali Zakaria, Jalpa Patel, Yazan Samhouri, Samira Ahsan, Lyle Goldman, Serge Sorser

**Affiliations:** ^1^Department of Internal Medicine, Ascension Providence Hospital, Michigan State University/College of Human Medicine, Southfield, MI, USA; ^2^Division of Gastroenterology, Ascension Providence Hospital, Michigan State University/College of Human Medicine, Southfield, MI, USA; ^3^Division of Hematology and Cellular Therapy, Allegheny Health Network Cancer Institute, Pittsburgh, PA, USA; ^4^Department of Hematology/Oncology, Ascension Providence Hospital, Michigan State University/College of Human Medicine, Southfield, MI, USA

## Abstract

Amyloidosis encompasses several diseases associated with deposition of low-molecular-weight proteins in an abnormal configuration. In light-chain amyloidosis (AL), monoclonal free lambda (*λ*) or kappa (*κ*) light chains are the amyloid proteins involved and can deposit in almost any organ. Symptoms vary depending on presence and extent of organ involvement, and thus, clinical presentation varies. Diagnosis requires biopsy of the affected tissue, and sometimes, fat pad or bone marrow biopsy is completed initially. Prognosis of AL amyloidosis depends on the presence of cardiac involvement. Treatment of AL amyloidosis involves systemic chemotherapy and evaluation for autologous stem cell transplant. Herein, we present a case report of an asymptomatic middle-aged female who was diagnosed with AL amyloidosis during an average-risk screening colonoscopy, which is an unusual setting. We discuss the workup involved, clinical presentation, and gastroenterology-related organ involvement.

## 1. Introduction

Amyloidosis refers to a broad group of diseases associated with extracellular deposition of protein fibrils characterized by beta-pleated sheet arrangement [1]. There are six known types of abnormal proteins associated with amyloidosis. The most common and most severe form is primary or light-chain-associated AL amyloidosis [1]. AA amyloidosis, also known as secondary amyloidosis (A refers to serum amyloid A protein (SAA), an acute phase reactant), has been described in inflammatory conditions. SAA is the protein that forms the amyloid fibril and deposits in the setting of chronic inflammatory states such rheumatoid arthritis and inflammatory bowel disease (IBD), with greater recognition in Crohn's disease (CD) [2]. Gastrointestinal (GI) manifestations are described in both AL amyloidosis and AA amyloidosis [3, 4]. Patients usually present with symptoms related to organ involvement; however, herein, we report a case of AL amyloidosis diagnosed incidentally during average-risk screening colonoscopy.

## 2. Case Presentation

**Figure 1:**
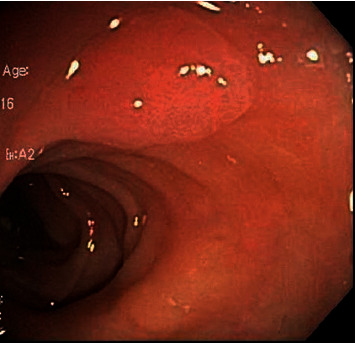
Colonoscopy showing a 10 mm sessile sigmoid colon polyp.

**Figure 2:**
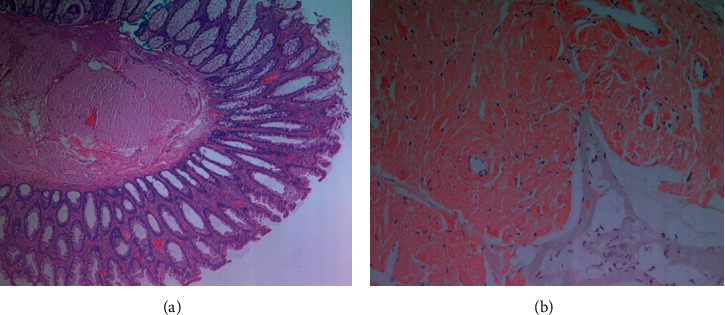
Sigmoid colon polypectomy showing benign colonic mucosa with hyperplastic changes and submucosal accumulation of eosinophilic materials, which are S100, CD117, CD34, and SMA negative (excluding neuroma, GIST, and leiomyoma) (a). The material is positive for Congo red stain consistent with amyloids (b).

A 67-year-old African American female with a medical history of hypertension, dyslipidemia, diabetes mellitus type II, and chronic kidney disease IIIa underwent age-appropriate average-risk screening colonoscopy which revealed one 10 mm sessile polyp (Paris II-a) in the sigmoid colon (Figure 1) [5]. Complete polypectomy was performed via hot snare, and histopathology showed colonic mucosa with hyperplastic changes, accompanied by submucosal accumulation of eosinophilic, paucicellular materials positive for Congo red stain, with green birefringence, consistent with amyloids (Figure 2). Two other 3 mm ascending colon tubular adenomas, mild sigmoid colon diverticulosis, and nonbleeding mild internal hemorrhoids were found.

She complained of intermittent dyspnea on exertion but otherwise had no other symptoms. She denied any abdominal pain, nausea, vomiting, hematochezia, weight loss, or change in bowel habits. She was referred to evaluate this incidentally diagnosed amyloidosis, and a follow-up abdominal fat pad biopsy confirmed amyloid vascular deposition. Further workup by hematology revealed a faint IgG kappa monoclonal band on serum immunofixation. The free kappa light chain was elevated at 29.3 mg/dl with a kappa/lambda ratio of 9.54. B2-microglobulin was 3.0 mg/L. Bone marrow biopsy showed plasma cell neoplasm (10%) with no amyloid deposition. The myeloma fluorescence in situ hybridization (FISH) panel was negative for the specific mutations, and cytogenetics showed a normal female karyotype. Amyloid typing with liquid chromatography (mass spectrometry) confirmed the diagnosis of kappa AL amyloidosis.

Further workup was done to evaluate for other organ (cardiac and renal) involvement of amyloidosis particularly with her symptoms of dyspnea and presence of CKD IIIa. Serum biomarkers showed a troponin-I level of 0.06 ng/mL (normal range: 0.00–0.10 ng/mL) and N-terminal pro-B-type natriuretic peptide (NT-proBNP) level of 320 pg/mL (normal range: 50–137 pg/mL). Cardiac catheterization revealed mild coronary artery disease, and cardiac MRI was negative for particular characteristics related to amyloid deposition. Kidney biopsy was not done as a 24-hour urine collection did not show substantial proteinuria/Bence Jones protein.

There was a multidisciplinary discussion with amyloidosis specialists regarding whether the patient should be treated with a systemic regimen. The consensus opinion was to monitor expectantly as the patient did not have renal or cardiac involvement. She remained asymptomatic, and the kappa light chain, renal function, and cardiac status remained stable. The timing of her surveillance colonoscopy was based on the two low-risk tubular adenomas as no guidance is available on the follow-up of benign polyps with amyloid deposition.

## 3. Discussion

Epidemiological data on amyloidosis are scarce; however, a recent large study by Quock et al. reported a mean age at diagnosis of 63, with higher incidence and prevalence in males [6]. These findings are comparable to previous studies. The reported incidence varies between the types of amyloidosis; one study showed 6.13 per million person-years for AL amyloidosis vs. 1.21 per million person-years for AA amyloidosis [7]. Moreover, kappa light chain amyloidosis is considered relatively rare in comparison to that of lambda light chain as the reported incidence is lower [8].

Amyloidosis can affect almost any organ. Common systemic manifestations include weakness, fatigue, purpura, and marked weight loss. Macroglossia is considered pathognomonic of the disease, but tissue confirmation is required, which is often obtained with abdominal fat pad biopsy. Although cardiac involvement is the leading cause of morbidity and mortality, involvement of the GI tract is not uncommon and can be associated with debilitating nonspecific symptoms and complications [9]. Localized disease to the GI tract is rare as systemic involvement is more common [9]. The prognosis of patients with AL amyloidosis and GI involvement is worse than that in those without known GI involvement. Moreover, if GI involvement is diagnosed, then there is a higher probability that other organs are involved and advanced disease is expected. Thus, a prompt evaluation is warranted [10].

Amyloid deposition can be present throughout the GI tract including almost all luminal organs with the greatest involvement reported in the small bowel [11]. Symptoms from small bowel involvement include diarrhea, constipation, and steatorrhea which are attributable to malabsorption and motility dysfunction [12]. Abdominal computed tomography (CT) scans may show edematous wall thickening of the small bowel, and endoscopic findings include bulk depositions causing mucosal protrusions and thickened intestinal folds of the small bowel [13, 14]. Nutritional deficiencies secondary to malabsorptive diarrhea can be an issue to some patients, and supportive therapy with multivitamin supplementation plays a role [15]. When the vasculature is affected, more life-threatening symptoms can develop such as hemorrhage, bowel infarction, perforation, and mesenteric ischemia [16]. Fortunately, pseudo-obstruction resulting from dysmotility is less commonly encountered, as prognosis is usually unpromising and surgery can be unsafe [17]. One report suggested that 8% of patients with amyloidosis have gastric involvement by biopsy, but only 1% exhibited symptoms. Symptoms range from nausea and vomiting to hematemesis and gastric outlet obstruction [1, 18]. Two large studies by Lee et al. and James et al. described similar findings of gastrointestinal manifestations related to amyloidosis; both showing motility-related predominant symptoms [19].

Hepatic involvement is common, but symptoms are usually mild with the most common findings being hepatomegaly and elevated alkaline phosphatase [20]. When present, ascites in amyloidosis is usually from cardiac failure and hypoalbuminemia rather than liver disease involvement [1]. GI manifestations are nonspecific; thus, high clinical suspicion coupled with detailed investigation is necessary to make the diagnosis. When untreated, AL amyloidosis is associated with a median 1-year mortality rate around 50%; thus, timely diagnosis is essential [20].

In AL amyloidosis, amyloids tend to deposit in the form of a mass in the submucosal and muscularis propria layers of the GI tract [21]. However, in AA amyloidosis, the protein fibrils tend to deposit in the lamina propria mucosae and submucosal layer in a macular or perivascular form [22]. Given these differences, polypoid protrusions and thickening of the valvulae conniventes are more common in AL amyloidosis, while fine mucosal appearance and friable mucosa are classic findings in AA amyloidosis [22, 23]. In addition, the disease occurs more frequently in the descending and rectosigmoid regions of the colon [24]. This pattern is similar to that seen in our patient who had sigmoid colonic polyps. Similar to gastrointestinal complaints, endoscopic features of AL amyloidosis are not specific to the disease. Multiple findings have been described including thickened folds, erosions, ulcerations, friability, and edema [19].

There are several cases describing colon involvement in amyloidosis. Fukui et al. described a similar case in a 72-year-old patient who was incidentally diagnosed with AL amyloidosis caused by monoclonal gammopathy of undetermined significance (MGUS); however, the patient had a positive fecal occult blood test which prompted the colonoscopy [25]. Axelrad et al. described a case of amyloid deposition throughout the colon with negative fat pad biopsy [9]. Lee et al. presented a case of AL amyloidosis in a patient diagnosed with ulcerative colitis (UC) [23]. Our case is different compared to the former cases; the patient had a positive fat biopsy and was not diagnosed with IBD. James et al. described a distinctive finding of submucosal hematomas positive for AL amyloidosis, in the setting of gastrointestinal bleeding [19]. This should prompt physicians to suspect amyloidosis when encountering submucosal hematomas during endoscopy.

Differentiation between types of amyloidosis is essential as controlling the underlying disorder is of paramount importance in AA amyloidosis, while AL amyloidosis requires treatment with chemotherapy and hematopoietic stem cell transplant. Remarkably, the mortality is high in patients receiving transplant and the morbidity seen is commonly secondary to GI complications including GI bleeding and toxic megacolon [26].

The diagnosis of AL amyloidosis can be challenging, and it is based on the Mayo Clinic and the International Myeloma Working Group criteria [27]. Our patient met the four criteria as she had GI tract involvement, positive amyloid staining with Congo red in fat aspirate, light-chain-related disease as mass spectrometry confirmed AL amyloidosis, and smoldering myeloma (presence of 10% plasma cells in the bone marrow with the absence of the CRAB criteria (hypercalcemia, renal insufficiency, anemia, and bone lesions)).

It is important to identify the extent of the disease as indications for systematic therapy are specific. Mostly all patients with systemic AL amyloidosis require treatment at the time of diagnosis [28]. However, in asymptomatic patients who are incidentally diagnosed, initial therapy can be delayed until the first sign of organ involvement develops [28]. The prognosis of AL amyloidosis in the setting of gastrointestinal and, specifically, colonic involvement is unknown; however, pseudo-obstruction of the colon carries a grave prognosis [1].

It is essential, yet a clinical dilemma, to establish organ involvement by amyloidosis. Consensus criteria revised in 2011 on amyloid and amyloidosis state that direct biopsy verification with symptoms is considered as “GI tract involvement,” while vascular only amyloid deposits biopsy verification without symptoms is not considered intestinal organ involvement [29]. Our patient had submucosal amyloid deposits but did not have any GI symptoms, which is why treatment was debatable and monitoring for symptoms or further organ involvement was the final plan. There is currently no established recommendation on the frequency of surveillance colonoscopy for polyps with amyloid deposition.

## 4. Conclusion

Endoscopic findings leading to a diagnosis of amyloidosis are uncommon; our patient is unique as she had no GI complaints, and her screening colonoscopy prompted the whole workup to be completed. If detected in an unusual setting, such as screening colonoscopy, it is important to refer patients for further evaluation as diagnosis can be time-sensitive. This case highlights the high-yield diagnostic value accompanying colonoscopy in diseases not necessarily belonging to the GI tract. It is important for gastroenterologists to consider amyloidosis amidst a background of nonspecific symptoms and refer patients to the appropriate subspecialist for further evaluation. There is currently no established recommendation on the frequency of surveillance colonoscopy for polyps with amyloid deposition.

## Data Availability

No data were used to support this study.
